# Polymorphonuclear leukocytes (PMNs) use different, strain-dependent mechanisms to kill the parasite *Trichomonas vaginalis*

**DOI:** 10.1128/mbio.03680-24

**Published:** 2025-06-26

**Authors:** Frances Mercer, Sandip Kumar Mukherjee, Chi-Lee Ho, Katherine Muratore, Patricia J. Johnson

**Affiliations:** 1 Department of Biological Sciences, California State Polytechnic University Pomona 6647 , Pomona, California, USA; 2 Department of Microbiology, Immunology and Molecular Genetics, University of California, Los Angeles, California, USA; University of California, Irvine, California, USA

**Keywords:** parasitology, immune cell killing, trogocytosis, NETosis, neutrophil

## Abstract

The unicellular parasite *Trichomonas vaginalis* (*Tv*) causes trichomoniasis, the most common non-viral sexually transmitted infection in humans. Trichomoniasis symptoms and sequelae vary substantially, and *in vitro* analyses have shown that strains differ extensively in their ability to infect and lyse cells of the urogenital tract. Polymorphonuclear leukocytes (PMNs) are known to kill *Tv*, yet to date, no studies have examined strain differences in PMN killing of this extracellular pathogen. Here, we have examined four strains of *Tv* for their susceptibility to killing by PMN trogocytosis, a process by which PMNs incrementally acquire fragments of the parasite’s plasma membrane leading to parasite death. Two strains were found to be rapidly and efficiently killed by trogocytosis, whereas the other two strains, although subjected to PMN trogocytosis, were resistant to killing. Strains that resist trogocytic killing were found to have an enhanced plasma membrane resealing capacity. Furthermore, these trogocytosis-resistant strains were susceptible to a later-stage killing mechanism that is significantly reduced in the presence of DNase, consistent with killing by neutrophil extracellular traps (NETosis). Together, our data reveal the use of multiple mechanisms for PMN killing of *Tv* and demonstrate strain-dependent resistance to rapid killing by trogocytosis, driven by membrane repair. Trogocytosis-resistant strains are subsequently killed by NETosis, likely circumventing the replacement of trogocytosis-sensitive strains by resistant strains over evolutionary time. Together, our data demonstrate that PMNs use different modalities to kill *Tv* and that *Tv* strains differ in their PMN-evasive properties.

**IMPORTANCE:**

*Trichomonas vaginalis* (*Tv*) causes the third most prevalent sexually transmitted infection globally and is the most common cause of vaginitis in the United States. Despite its prevalence, little is known about how the human immune system combats and kills *Tv*. We have previously shown that the abundant white blood cells called polymorphonuclear leukocytes (PMNs), known to kill pathogens using multiple mechanisms, kill *Tv* using a rapid-killing mechanism called trogocytosis. Here, we examined whether *Tv* clinical isolates with different pathogenic properties are killed by PMNs using other mechanisms. We demonstrate that clinical isolates that are resistant to trogocytic killing are better at plasma membrane repair and are efficiently killed by a late-stage mechanism called NETosis. Thus, PMN uses multiple, strain-specific mechanisms to kill *Tv* isolates with varying virulence properties. This study sheds light on host cell killing mechanisms that may impact pathogenic outcomes and sequelae during *Tv* infection.

## OBSERVATION

*Trichomonas vaginalis (Tv*) is a unicellular parasite that causes the third most common sexually transmitted infection (STI) in the United States and globally (1). Polymorphonuclear leukocytes (PMNs) are home to the site of *Tv* infection in females (2, 3) and are capable of potent and rapid parasite destruction *in vitro* (2–6). We previously established an *in vitro* model of PMN interaction with *Tv*, using primary PMNs isolated from fresh human blood and pooled human serum, and determined that human PMNs can kill *Tv* using trogocytosis (trogo = to nibble), a contact-dependent process in which PMNs actively acquire fragments of the parasite’s plasma membrane in a piece-meal fashion until the parasite dies (5). Upon PMN-parasite co-culture, trogocytosis commences almost immediately after cell-cell contact, and most parasites are killed within 10 min (5). Trogocytosis is a rapid killing mechanism (3, 7), in contrast to neutrophil extracellular trap (NET)osis which is activated hours after pathogen encounter and is hypothesized to be a last-ditch, suicidal effort to kill pathogens that have evaded PMN rapid killing mechanisms (8). PMNs were recently shown to release NETs in response to *Tv* encounter, limiting the subsequent growth of the parasites in culture (9), suggesting the utility of NETs in *Tv* killing.

**Fig 1:**
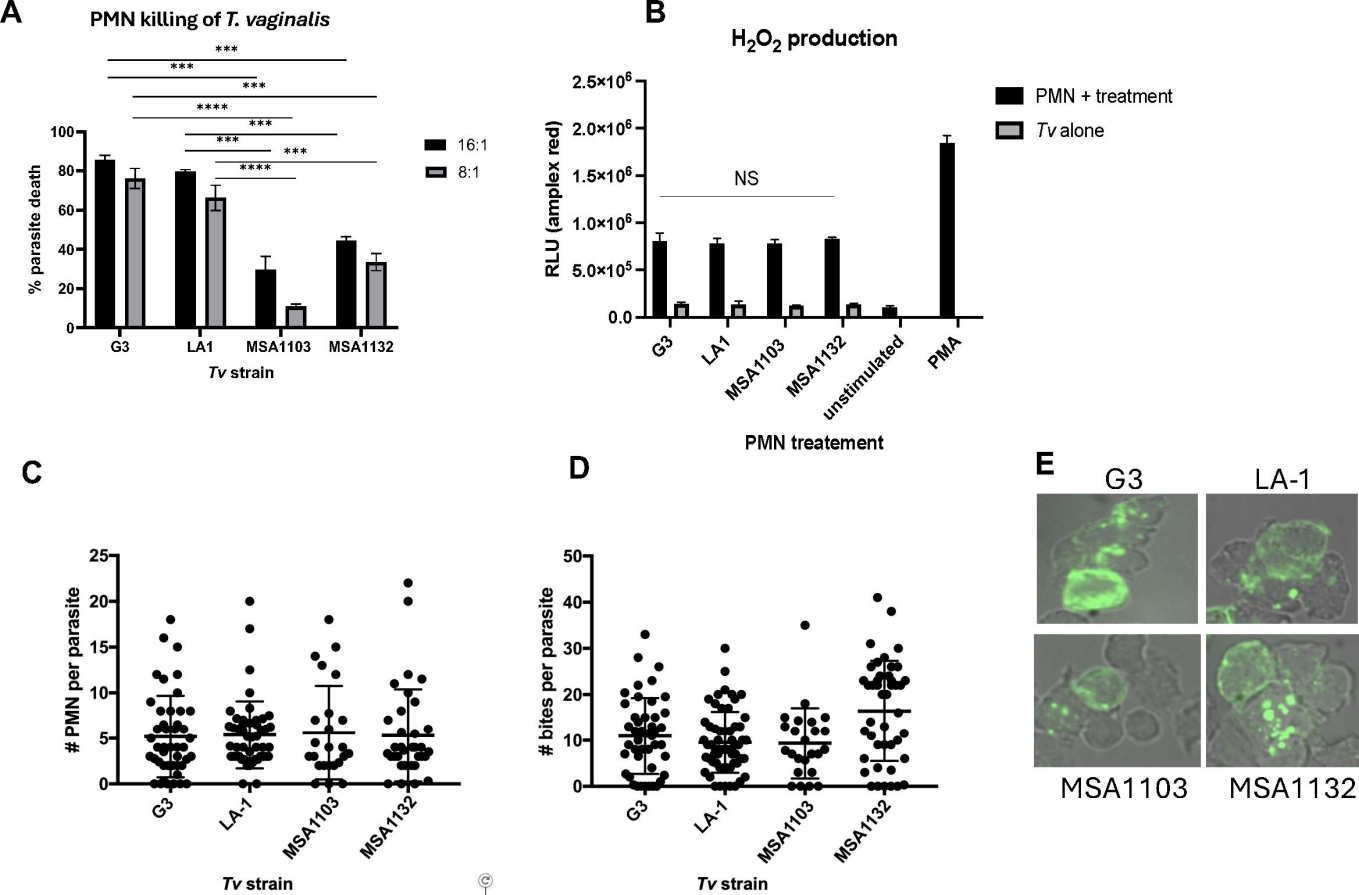
All *Trichomonas vaginalis* (*Tv*) strains activate and are swarmed and trogocytosed by PMNs, but not all are equally susceptible to rapid killing. (**A**) Four *Tv* strains (G3, LA-1, MSA1103, and MSA1132) and human blood donor-derived PMNs were pre-labeled in Cell Tracker Red or CFSE, respectively, and co-cultured at the indicated ratios in u-bottom plates for 1 h (5). Percent parasite death was determined by comparing the count of surviving parasites in PMN co-culture conditions to the count of parasites in parasite-alone control wells, using a BD LSR2 flow cytometer. The percent of parasites killed by PMNs is shown for each strain. PMNs were isolated as described in Mercer et al. (5). Human blood material was obtained from the University of California, Los Angeles, CFAR Core Facility. See Mercer et al. (5) for the gating scheme used for this experiment. (**B**) Hydrogen peroxide production by PMNs was measured as a proxy for PMN activation using the Amplex Red extracellular H_2_O_2_ detection kit (Invitrogen). Activation was assessed by relative light units (RLUs) detected after co-culture with different strains of *Tv* at a ratio of 8:1 PMN:*Tv* or with a positive control (100 nM PMA). Data for panels **A** and **B** are the average of three independent experiments each performed in triplicate. Paired *t*-tests were performed as indicated (***, <0.001; ****, <0.0001). Error bars represent standard deviation (SD). The number of PMN surrounding parasites of the four strains (**C**) and the number of membrane fragments acquired from the parasite (“bites”) (**D**) was assessed by labeling parasites with EZ-link Biotin (Thermo) and fluorescently conjugated streptavidin and seeding onto lawns on PMNs on poly-L-lysine coated coverslips inserted into tissue culture plates. After washing and fixation with 4% PFA, coverslips were imaged using fluorescent microscopy. All slides were blinded before imaging and quantifying images, with strain identities revealed after data were compiled and analyzed. Data shown in panels **C** and **D** are aggregates of parasites counted in three independent experiments with unique donors’ PMN, with each strain tested at least twice, and at least 10 parasites visualized and quantified each time. Each of the three independent experiments was performed in triplicate, using a PMN:Tv ratio of 8:1. Paired *t*-tests were performed, and there was no statistical difference in the number of PMN swarming or the number of bites taken (C and D) for the four strains. (**E**) Representative images used to quantify swarming (**C**) and parasite bites (**D**).

Here, we have tested the ability of human PMNs to kill four phenotypically variable *Tv* strains (10). Using a cytotoxicity assay to measure PMN killing (5), we found that *Tv* strains G3 and LA1 were efficiently killed at >70% at a PMN:*Tv* ratio of 8:1 after 1 h. In contrast, for strains MSA1103 and MSA1132, only ~10% and ~33%, respectively, were killed at the same PMN:*Tv* ratio (Fig. 1A). To test whether PMNs do not get activated as efficiently by strains MSA1103 and MSA1132, as they are by strains G3 and LA1, we compared the induction of reactive oxygen species by-product hydrogen peroxide (H_2_O_2_) by PMNs in the presence of the four strains. We found that all strains induced equal amounts of H_2_O_2_ production (Fig. 1B), indicating that PMN is activated equally well by the four strains.

Trogocytic killing requires cell-cell contact between PMNs and *Tv* (5), and PMNs follow secreted cues from both parasites and other PMNs to home to individual parasites in swarms (5, 11, 12). We, therefore, tested whether PMNs exhibit reduced swarming activity toward strains resistant to killing, compared to sensitive strains, by co-culturing PMNs and *Tv* on coverslips and counting the number of PMNs in cellular aggregates around each parasite (swarms) after 10 min, using confocal microscopy. On average, five PMNs were found to surround each parasite for all strains tested (Fig. 1C), demonstrating that PMNs are able to localize to and establish contact with resistant strains as efficiently as with sensitive strains. In the same imaging assays, we quantified trogocytosis of each parasite strain and unexpectedly found that resistant strains were still trogocytosed as efficiently as susceptible strains. On average, the number of *Tv* membrane fragments (“bites”) acquired by PMNs from each parasite was ~10 for strains G3, LA1, and MSA1103, and even higher, at ~15 “bites”, for MSA1132, which is resistant to rapid killing (Fig. 1D). We, therefore, concluded that neither a reduction in PMN swarming nor trogocytosis of resistant strains was responsible for the reduced PMN killing of resistant strains. Hence, we classified the resistant strains as trogocytosis-resistant, since they survive trogocytosis.

**Fig 2:**
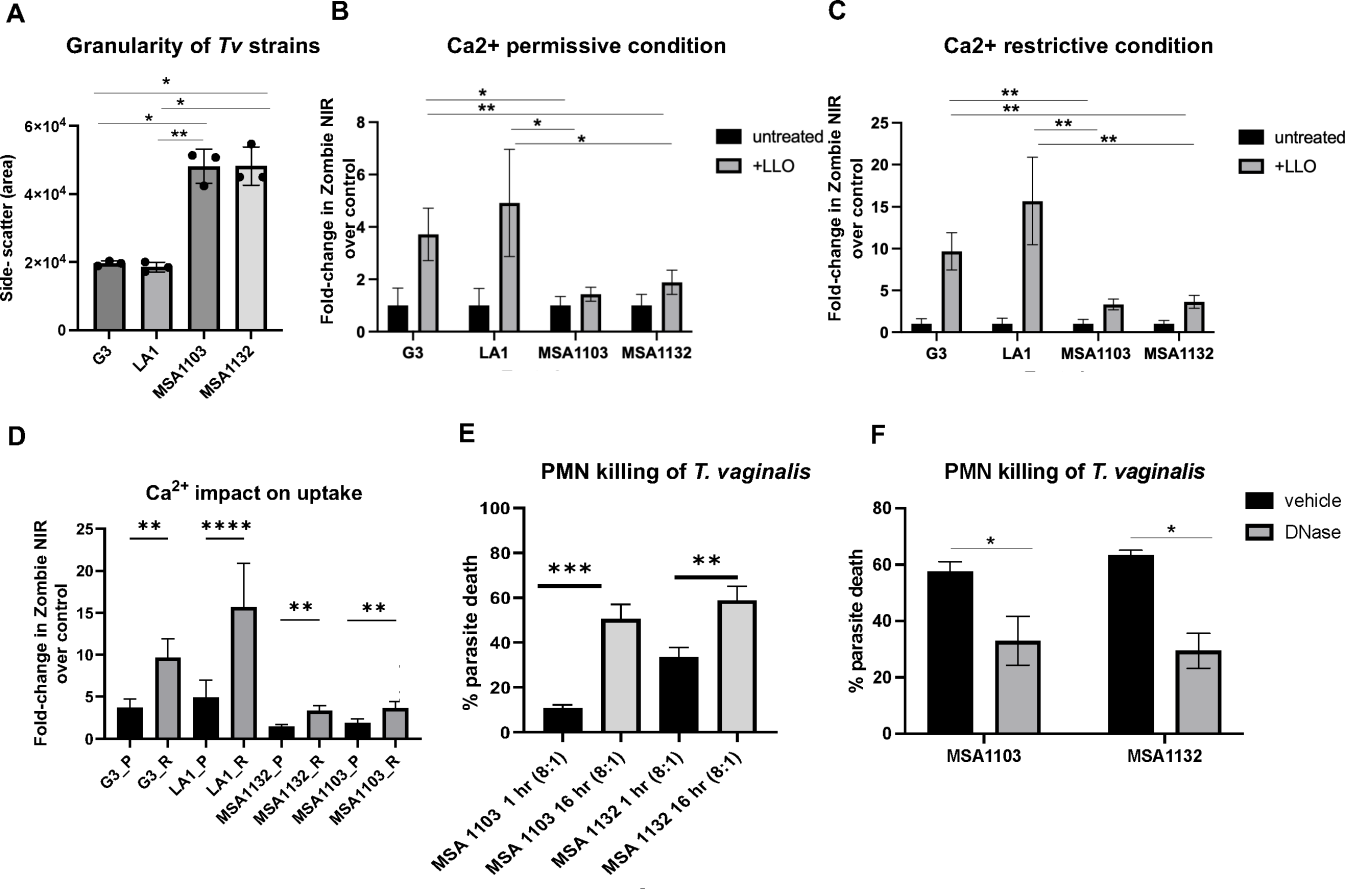
*Tv* strains resistant to killing by trogocytosis have increased cellular granularity, internalize less dye than sensitive strains upon listeriolysin O-induced plasma membrane breach, and are efficiently killed upon prolonged exposure to PMN. (**A**) All four *Tv* strains were subjected to flow cytometry, and the ssc-A (side-scatter, area) was assessed. Data are an average of three independent experiments each performed in triplicate. Paired *t*-tests were performed as indicated (*, <0.05; **, <0.01; ***, <0.001; ****, <0.0001). (B and C) Trogocytosis-sensitive strains (G3 and LA-1) and trogocytosis-resistant strains (MSA1103 and MSA1132) were collected and washed with Hank’s Balanced Salt Solution (HBSS), followed by washing with M1 buffer (HBSS, 500 µM MgCl_2_, 1.2 mM CaCl_2_, 10 mM HEPES, and 25 mM glucose) and resuspended in M1 buffer for parasites tested using Ca^2+^ permissive conditions. Parasites tested using restrictive conditions lacking Ca^2+^ were instead washed with M2-EGTA buffer (HBSS, 500 µM MgCl_2_, 10 mM HEPES, 25 mM glucose, and 5 µM EGTA) followed by one wash with M2 buffer (HBSS, 500 µM MgCl_2_, 10 mM HEPES, and 25 mM glucose) and resuspended in M2 buffer. Parasites were subsequently incubated with 10 nM listeriolysin O (Abcam) and Zombie NIR dye (Biolegend) at 1:500, in their respective media on ice for 5 min followed by 20 min at RT. Parasites incubated in either M1 or M2 buffer with Zombie NIR dye, without the addition of listeriolysin O, were included as negative controls. Parasites were then washed with PBS, fixed with 4% PFA, and analyzed on a BD LSRII flow cytometer to measure the amount of Zombie NIR that accumulated in sensitive and resistant parasites under permissive (plus Ca^2+^) and restrictive (minus Ca^2+^) conditions. (**D**) Data shown in panels B and C expressed as fold change in Zombie NIR dye internalization normalized to controls without the addition of LLO. Fold change was normalized by dividing the amount of Zombie NIR dye internalized in the presence of LLO by that internalized under the same conditions without the addition of LLO. (**E**) Trogocytosis-resistant strains MSA 1103 and MSA 1132 pre-labeled in Cell Tracker Red were incubated with PMNs from the same donor, labeled with CFSE, and co-cultured for either 1 or 16 h at an 8:1 PMN:*Tv* ratio. Parasite death at each time point was then determined as described in Fig. 1 legend. (**F**) Pre-labeled *Tv* and primary human neutrophils (PMNs) were co-cultured in a cytotoxicity assay at an 8:1 PMN:*Tv* ratio for 16 h, in the absence and presence of DNase1 100 µ/mL or vehicle control (HBSS). The percent of parasites killed by PMNs is shown for each strain. Data for panels **A–E** are an average of three independent experiments each performed in triplicate. Paired *t*-tests were performed as indicated (*, <0.05; **, <0.01). All error bars represent standard deviation (SD).

The data above led us to propose that trogocytosis-resistant strains may be able to survive trogocytosis by more efficiently repairing plasma membrane breaches using conserved mechanisms that mobilize cytosolic vesicles to the plasma membrane, where vesicle fusion and subsequent endocytosis of damaged membrane maintains cell integrity (13). With the prediction that cells with greater capacity for rapid membrane repair may be richer in vesicles, we then analyzed the side scatter, a measure of internal cell complexity, or granularity (14), of all four strains using flow cytometry. We found that trogocytosis-resistant strains had ~2-fold higher side-scatter values than trogocytosis-sensitive strains (Fig. 2A; Fig. S1), indicating resistant strains may be more poised to repair membrane breaches more efficiently.

To directly test the ability of the different *Tv* strains to repair membrane breaches, we exposed the strains to the pore-forming toxin listeriolysin O (LLO) used in membrane repair assays (15) and compared the uptake of Zombie NIR dye in the presence (permissive) or absence of calcium (restrictive) to assess membrane resealing (16). Zombie NIR dye is a membrane-impermeant amine-reactive dye that can only pass into cells through breaches in the plasma membrane. We found that in the presence of calcium, trogocytosis-resistant strains MSA1103 and MSA1132 took up less Zombie NIR dye than trogocytosis-sensitive strains G3 and LA-1 when treated with an equivalent amount of LLO (Fig. 2B; Fig. S2), consistent with a more rapid resealing of membrane pores by resistant strains MSA1103 and MSA1132. As membrane resealing is a calcium-dependent process (17), we also tested whether *Tv* strains took up more Zombie NIR dye when the assay was performed in the absence of calcium (Fig. 2C). As predicted, we found that more dye was internalized by *Tv* in the absence of calcium (restricted conditions) compared to *Tv* in the presence of calcium (permissive conditions) (Fig. 2D; Fig. S2). Figure 2D shows the fold change of Zombie NIR MFI in permissive conditions and restrictive conditions normalized to controls without the addition of LLO and demonstrates significantly greater LLO uptake for all strains in the absence of calcium. These data support a model of calcium-dependent resealing of the plasma membrane of *Tv* upon trogocytic attack by PMNs and indicate that *Tv* strains that survive trogocytosis are better at resealing their plasma membranes to evade killing.

To determine whether trogocytosis-resistant strains are efficiently killed upon prolonged incubation with PMN, as opposed to the 1 h incubation used in our rapid killings assay, and whether killing is dependent on NETosis, we compared the killing of MSA1103 and MSA1132 strains by PMNs after incubation for either 1 and 16 hours. The percent killing of MSA1103 increased from 11% at 1 h to 51% at 16 h and the percent killing of MSA1132 increased from 33% at 1 h to 59% at 16 h (Fig. 2E), demonstrating that trogocytosis-resistant strains are more efficiently killed upon prolonged exposure to PMNs. To test whether this delayed killing was dependent on DNA extracellular traps, parasite death was then assessed after 16 h in the absence or presence of DNase I, known to degrade NETs (18). We found that ~60% of parasites were killed in the absence of DNase I, whereas the addition of DNase I reduced killing to ~30% (Fig. 2F). We, therefore, concluded that trogocytosis-resistant strains are susceptible to PMN killing *in vitro* by mechanisms that include NETosis. It is notable that trogocytosis-resistant strains killed by NETosis are still more resistant to killing than trogocytosis-sensitive strains. This is likely because all strains used in this study harbor the bacterial symbiont *Mycoplasma hominis,* and we have found that the symbiont protects parasite killing by NETosis, but not by trogocytosis (our unpublished results), as predicted by Cacciotto et al. (19).

In summary, we show that *Tv* strains vary in their susceptibility to trogocytic killing by PMNs, with strains that are resistant to trogocytosis demonstrating enhanced plasma membrane resealing capacity. We also show that strains resistant to trogocytic killing are susceptible to killing by NETosis, demonstrating that PMN uses multiple methods to attack and kill trichomonads.

## SUPPLEMENTAL MATERIAL

Supplemental Figure Legends mbio.03680-24-s0001.docx Fig. S1 and S2 legends.

Fig. S1 mbio.03680-24-s0002.tiff Gating scheme used for Fig. 2A.

Fig. S2 mbio.03680-24-s0003.tiff Gating scheme used for Fig. 2E and F.

ASM does not own the copyrights to Supplemental Material that may be linked to, or accessed through, an article. The authors have granted ASM a non-exclusive, world-wide license to publish the Supplemental Material files. Please contact the corresponding author directly for reuse.
