# Supplementary material for: Polymorphonuclear leukocytes (PMNs) use different, strain-dependent mechanisms to kill the parasite Trichomonas vaginalis
Source: mBio. 2025 Jun 26;16(8):e03680-24. doi: 10.1128/mbio.03680-24 (PMC12345268; doi:10.1128/mbio.03680-24)
Supplement: Supplemental Figure Legends — Fig. S1 and S2 legends. [file mbio.03680-24-s0001.docx]

**Supplemental Information**

**Figure S1: Gating scheme used for Figure 2A**

To generate bar-graphs in Fig2A, each sample was gated on intact cells, based on size and granularity. The intact cell gate was then analyzed for the side-scatter area (SSC-A). Representative histograms of triplicate samples for each strain are shown.

**Figure S2: Gating scheme used for Figure 2E-F**

To generate bar-graphs in Fig2E-F, each sample was gated on intact cells, based on size and granularity. The intact cell gate was then analyzed for the mean-fluorescence intensity of the NIR channel (Zombie NIR). Representative histograms show Zombie NIR mean-fluorescence intensity for strain G3 derived from three independent experiments.
