# Supplementary figures and images for: Polymorphonuclear leukocytes (PMNs) use different, strain-dependent mechanisms to kill the parasite Trichomonas vaginalis
Source: mBio. 2025 Jun 26;16(8):e03680-24. doi: 10.1128/mbio.03680-24 (PMC12345268; doi:10.1128/mbio.03680-24)

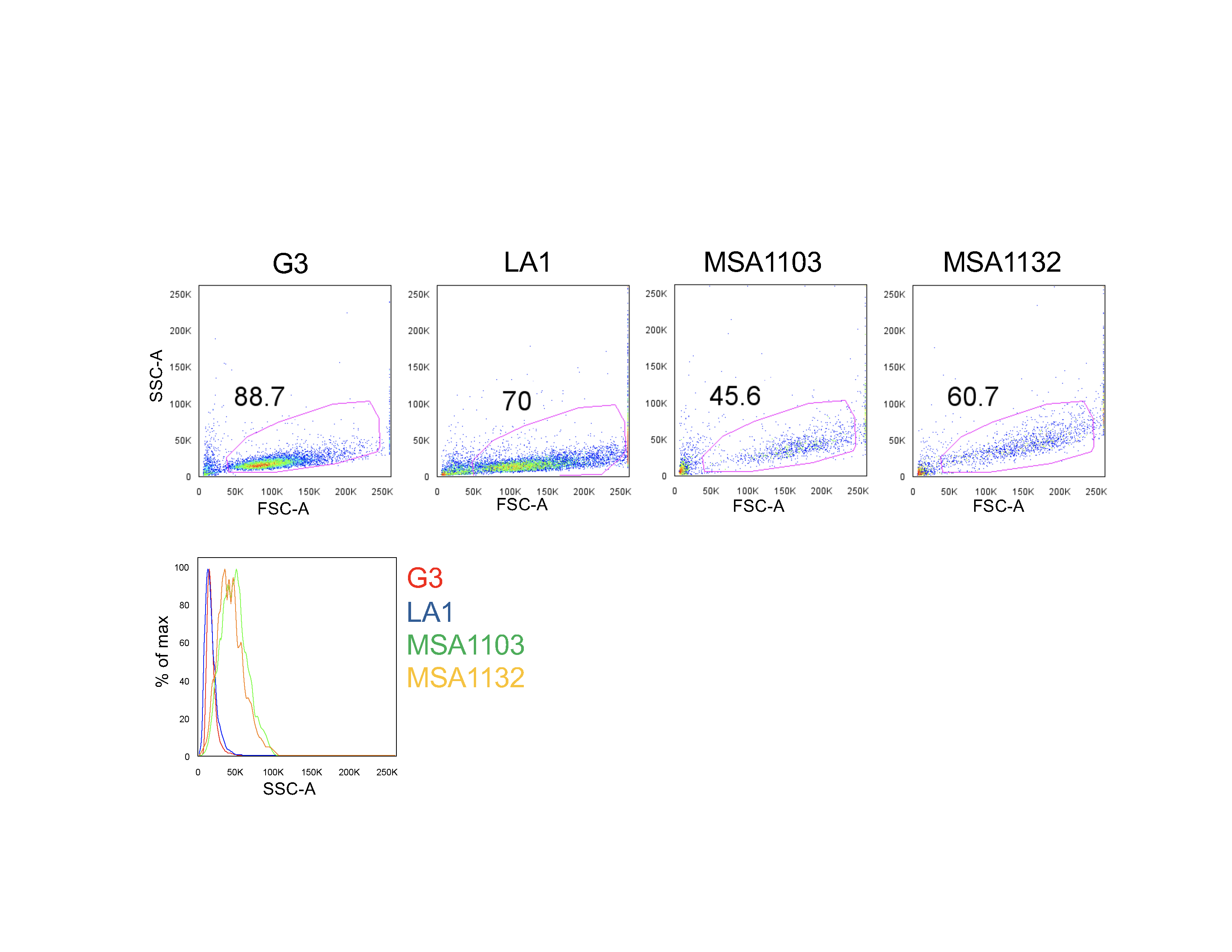

Supplement: Fig. S1 — Gating scheme used for Fig. 2A. [file mbio.03680-24-s0002.tiff]

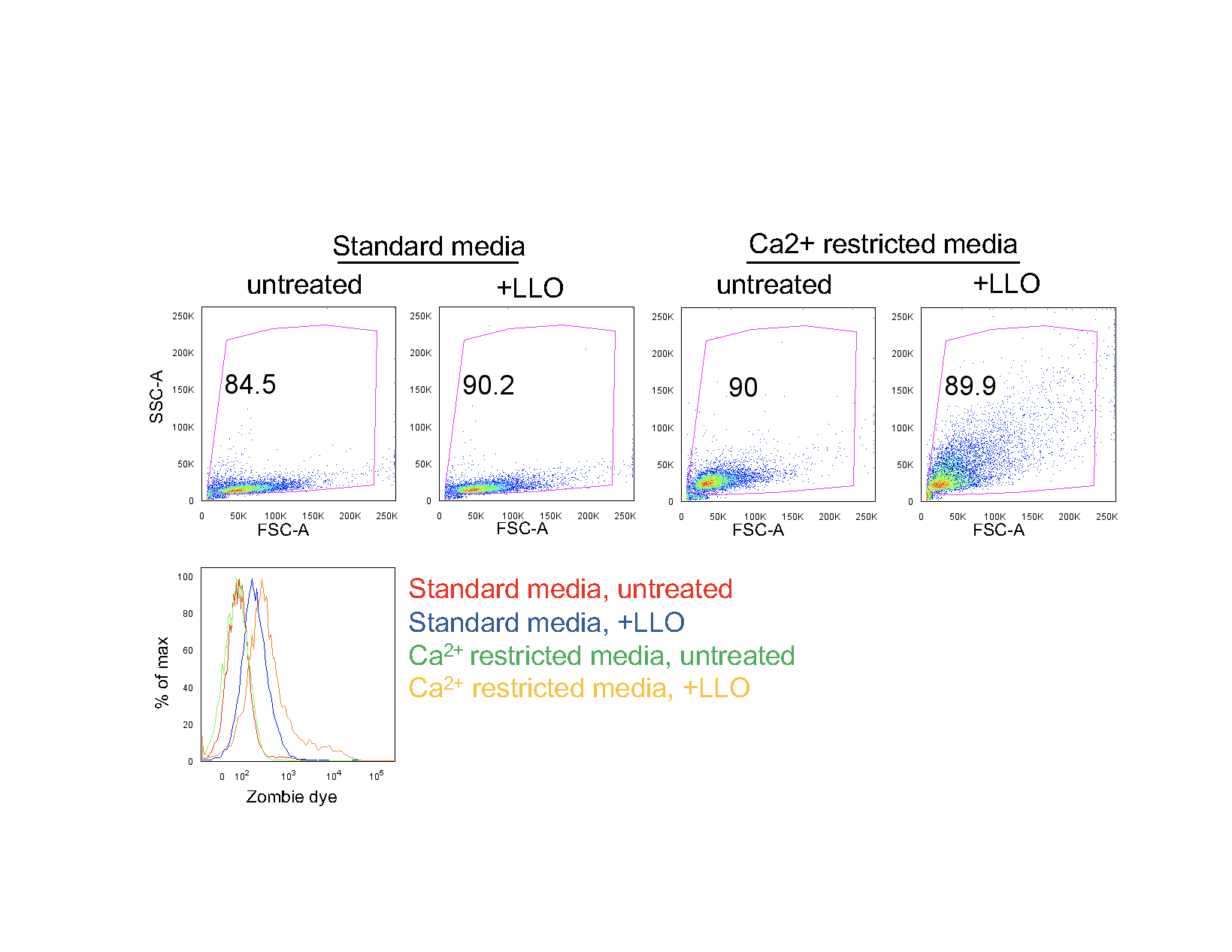

Supplement: Fig. S2 — Gating scheme used for Fig. 2E and F. [file mbio.03680-24-s0003.tiff]
